# Tetra­kis{μ_3_-2-[(2-hy­droxy­eth­yl)amino]­ethano­lato}tetra­kis­[chloridonickel(II)] methanol solvate

**DOI:** 10.1107/S1600536810024384

**Published:** 2010-06-26

**Authors:** Youzhu Yu, Yuhua Guo, Lei Lv, Dacheng Li

**Affiliations:** a Department of Chemistry and Environmental Engineering, Anyang Institute of Technology, Henan 455000, People’s Republic of China; b College of Chemistry and Chemical Engineering, Liaocheng University, Shandong 252059, People’s Republic of China

## Abstract

The complex mol­ecule of the title compound, [Ni_4_(C_4_H_10_NO_2_)_4_Cl_4_]·CH_3_OH, consists of a cubane-like {Ni_4_O_4_} core in which each nickel(II) atom is six-coordinated in a distorted octa­hedral geometry by one N and four O atoms of three mono-deprotonated diethano­lamine ligands and by a chloride anion. The mol­ecular conformation is stabilized by intra­molecular O—H⋯Cl bonds. In the crystal structure, complex mol­ecules and methanol solvent mol­ecules are linked into a three-dimensional network by N—H⋯Cl, N—H⋯O and O—H⋯Cl hydrogen-bonding inter­actions.

## Related literature

For the magnetic properties and structures of related compounds, see: Cadiou *et al.* (2001 ▶); Ferguson *et al.* (2008 ▶).
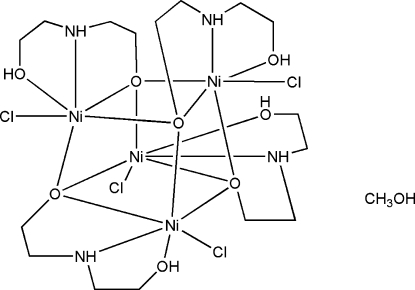


## Experimental

### 

#### Crystal data

[Ni_4_(C_4_H_10_NO_2_)_4_Cl_4_]·CH_4_O
*M* *_r_* = 825.20
Triclinic, 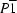

*a* = 10.8244 (12) Å
*b* = 11.5609 (13) Å
*c* = 13.2797 (17) Å
α = 91.741 (1)°
β = 91.845 (1)°
γ = 111.283 (2)°
*V* = 1546.1 (3) Å^3^
*Z* = 2
Mo *K*α radiation
μ = 2.79 mm^−1^
*T* = 298 K
0.39 × 0.25 × 0.15 mm

#### Data collection

Bruker SMART 1000 CCD diffractometer
Absorption correction: multi-scan (*SADABS*; Sheldrick, 1996 ▶) *T*_min_ = 0.409, *T*_max_ = 0.680
8045 measured reflections
5352 independent reflections
3889 reflections with *I* > 2σ(*I*)
*R*_int_ = 0.025

#### Refinement

*R*[*F* ^2^ > 2σ(*F* ^2^)] = 0.043
*wR*(*F* ^2^) = 0.141
*S* = 1.01
5352 reflections
344 parameters
H-atom parameters constrained
Δρ_max_ = 1.01 e Å^−3^
Δρ_min_ = −0.74 e Å^−3^

### 

Data collection: *SMART* (Siemens, 1996 ▶); cell refinement: *SAINT* (Siemens, 1996 ▶); data reduction: *SAINT*; program(s) used to solve structure: *SHELXS97* (Sheldrick, 2008 ▶); program(s) used to refine structure: *SHELXL97* (Sheldrick, 2008 ▶); molecular graphics: *SHELXTL* (Sheldrick, 2008 ▶); software used to prepare material for publication: *SHELXTL*.

## Supplementary Material

Crystal structure: contains datablocks I, global. DOI: 10.1107/S1600536810024384/rz2468sup1.cif

Structure factors: contains datablocks I. DOI: 10.1107/S1600536810024384/rz2468Isup2.hkl

Additional supplementary materials:  crystallographic information; 3D view; checkCIF report

## Figures and Tables

**Table 1:** Hydrogen-bond geometry (Å, °)

*D*—H⋯*A*	*D*—H	H⋯*A*	*D*⋯*A*	*D*—H⋯*A*
O4—H4⋯Cl2	0.82	2.27	3.087 (4)	176
O8—H8⋯Cl3	0.82	2.19	3.013 (4)	179
O2—H2⋯Cl4	0.82	2.24	3.058 (4)	175
O6—H6⋯Cl2	0.82	2.60	3.346 (4)	153
N1—H1⋯Cl1^i^	0.91	2.62	3.476 (5)	157
N3—H3⋯Cl2^ii^	0.91	2.62	3.448 (5)	152
N4—H4*AA*⋯O9	0.91	2.52	3.228 (9)	135
N2—H2*AA*⋯O9^iii^	0.91	2.18	3.060 (10)	162
O9—H9⋯Cl4	0.82	2.76	3.224 (7)	118

Symmetry codes: (i) 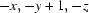; (ii) 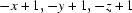; (iii) 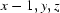.

**Fig. 1.**
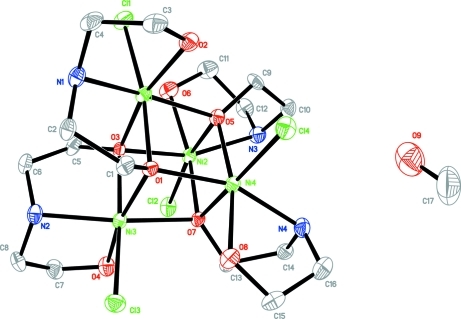
The molecular structure of the title compound, with atom labels and 30% probability displacement ellipsoids. Hydrogen atoms are omitted for clarity.

## supplementary crystallographic information

### Comment

The potential of the nickel(II) was highlighted when a few Ni-containing complexes have been reported for their 'single molecule magnetism' (SMM) behaviour. (Cadiou *et al.*, 2001). We report here the synthesis and structure of the title compound.

In the title complex (Fig. 1), each nickel(II) metal atom is six-coordinated by three µ_3_-O donors derived from the alkoxide groups of three mono-deprotonated diethanolamine ligands, one hydroxy O and one amine N atom of the same ligand, and by a Cl anion, forming a distorted octahedron. Bond lengths and angles are typical and are comparable with those observed in the related acetonitrile solvate octadecahydrate complexes (Ferguson *et al.*, 2008). The conformation of the complex molecule is stabilized by intramolecular O—H···Cl bonds (Table 1). In the crystal structure, complex molecules and solvent molecules are linked through intermolecular N—H···Cl, N—H···O and O—H···Cl hydrogen bonds (Table 1) into a three-dimensional network.

### Experimental

To a stirred methanol solution (15 ml) of NiCl_2_.6H_2_O (2 mmol, 475 mg) was added diethanolamine (4 mmol, 421 mg) in 5 ml methanol. After 10 min NaOAc was added and the mixture stirred for 6 h. The resulting blue solution was filtrated and was allowed to stand at room temperature for about one week, whereupon blue block crystals suitable for X-ray diffraction analysis were obtained.

### Refinement

All H atoms were placed in geometrically idealized positions and treated as riding on their parent atoms, with C—H = 0.96-0.97 Å, N—H = 0.91 Å, O—H = 0.82 Å, and with *U*_iso_(H) = 1.2*U*_eq_(C, N) or 1.5*U*_eq_(C, O) for methyl and hydroxy H atoms.

### Crystal data

**Table:**

[Ni_4_(C_4_H_10_NO_2_)_4_Cl_4_]·CH_4_O	*Z* = 2
*M**_r_* = 825.20	*F*(000) = 852
Triclinic, *P*1	*D*_x_ = 1.773 Mg m^−^^3^
Hall symbol: -P 1	Mo *K*α radiation, λ = 0.71073 Å
*a* = 10.8244 (12) Å	Cell parameters from 2996 reflections
*b* = 11.5609 (13) Å	θ = 2.4–28.3°
*c* = 13.2797 (17) Å	µ = 2.79 mm^−^^1^
α = 91.741 (1)°	*T* = 298 K
β = 91.845 (1)°	Block, blue
γ = 111.283 (2)°	0.39 × 0.25 × 0.15 mm
*V* = 1546.1 (3) Å^3^	

### Data collection

**Table:**

Bruker SMART 1000 CCD diffractometer	5352 independent reflections
Radiation source: fine-focus sealed tube	3889 reflections with *I* > 2σ(*I*)
graphite	*R*_int_ = 0.025
phi and ω scans	θ_max_ = 25.0°, θ_min_ = 1.5°
Absorption correction: multi-scan (*SADABS*; Sheldrick, 1996)	*h* = −8→12
*T*_min_ = 0.409, *T*_max_ = 0.680	*k* = −13→13
8045 measured reflections	*l* = −13→15

### Refinement

**Table:**

Refinement on *F*^2^	Primary atom site location: structure-invariant direct methods
Least-squares matrix: full	Secondary atom site location: difference Fourier map
*R*[*F*^2^ > 2σ(*F*^2^)] = 0.043	Hydrogen site location: inferred from neighbouring sites
*wR*(*F*^2^) = 0.141	H-atom parameters constrained
*S* = 1.01	*w* = 1/[σ^2^(*F*_o_^2^) + (0.085*P*)^2^] where *P* = (*F*_o_^2^ + 2*F*_c_^2^)/3
5352 reflections	(Δ/σ)_max_ = 0.001
344 parameters	Δρ_max_ = 1.01 e Å^−^^3^
0 restraints	Δρ_min_ = −0.74 e Å^−^^3^

### Fractional atomic coordinates and isotropic or equivalent isotropic displacement parameters (Å^2^)

**Table:**

	*x*	*y*	*z*	*U*_iso_*/*U*_eq_	
Ni1	0.28194 (7)	0.64164 (6)	0.10224 (5)	0.0256 (2)	
Ni2	0.35928 (7)	0.55768 (6)	0.30672 (5)	0.0258 (2)	
Ni3	0.25126 (7)	0.77201 (6)	0.31177 (5)	0.0250 (2)	
Ni4	0.52684 (7)	0.82657 (6)	0.22639 (5)	0.0252 (2)	
Cl4	0.65383 (15)	0.88748 (14)	0.08011 (11)	0.0391 (4)	
Cl1	0.17830 (15)	0.43190 (14)	0.03338 (12)	0.0399 (4)	
Cl3	0.30181 (15)	0.98516 (14)	0.36147 (11)	0.0381 (4)	
Cl2	0.28239 (14)	0.49380 (14)	0.47358 (11)	0.0375 (4)	
O1	0.3416 (3)	0.8145 (3)	0.1730 (3)	0.0234 (8)	
O3	0.2025 (3)	0.5972 (3)	0.2474 (3)	0.0238 (8)	
O7	0.4270 (3)	0.7491 (3)	0.3498 (2)	0.0228 (8)	
O5	0.4480 (3)	0.6348 (3)	0.1788 (3)	0.0252 (8)	
O4	0.1916 (4)	0.7173 (4)	0.4608 (3)	0.0370 (10)	
H4	0.2120	0.6559	0.4656	0.055*	
O8	0.5626 (4)	1.0166 (4)	0.2700 (3)	0.0379 (10)	
H8	0.4914	1.0069	0.2950	0.057*	
O2	0.4041 (4)	0.6984 (4)	−0.0261 (3)	0.0351 (10)	
H2	0.4681	0.7497	0.0051	0.053*	
N1	0.1626 (5)	0.7099 (4)	0.0125 (3)	0.0319 (11)	
H1	0.0760	0.6598	0.0164	0.038*	
N3	0.5163 (5)	0.4904 (4)	0.3159 (4)	0.0346 (12)	
H3	0.5644	0.5204	0.3747	0.042*	
C2	0.1813 (5)	0.8351 (5)	0.0534 (4)	0.0330 (14)	
H2A	0.1120	0.8292	0.0997	0.040*	
H2B	0.1725	0.8856	−0.0014	0.040*	
C13	0.5074 (5)	0.7992 (5)	0.4385 (4)	0.0304 (13)	
H13A	0.4723	0.7465	0.4942	0.036*	
H13B	0.5065	0.8811	0.4553	0.036*	
O6	0.2621 (4)	0.3722 (4)	0.2390 (3)	0.0406 (10)	
H6	0.2429	0.3795	0.2975	0.061*	
C10	0.6016 (6)	0.5390 (6)	0.2315 (5)	0.0361 (14)	
H10A	0.6386	0.4784	0.2089	0.043*	
H10B	0.6746	0.6141	0.2544	0.043*	
C9	0.5254 (6)	0.5683 (5)	0.1428 (4)	0.0302 (13)	
H9A	0.5875	0.6174	0.0955	0.036*	
H9B	0.4684	0.4916	0.1080	0.036*	
C1	0.3169 (5)	0.8993 (5)	0.1086 (4)	0.0281 (13)	
H1A	0.3857	0.9264	0.0601	0.034*	
H1B	0.3174	0.9718	0.1476	0.034*	
C16	0.7654 (6)	1.0042 (6)	0.3340 (5)	0.0452 (17)	
H16A	0.8152	1.0312	0.2741	0.054*	
H16B	0.8278	1.0264	0.3919	0.054*	
C8	−0.0011 (6)	0.7483 (6)	0.3952 (5)	0.0368 (15)	
H8A	−0.0974	0.7151	0.3936	0.044*	
H8B	0.0290	0.8364	0.4134	0.044*	
N4	0.6958 (5)	0.8668 (5)	0.3262 (4)	0.0351 (12)	
H4AA	0.7511	0.8319	0.2995	0.042*	
N2	0.0443 (4)	0.7305 (4)	0.2953 (3)	0.0320 (11)	
H2AA	0.0296	0.7855	0.2535	0.038*	
C12	0.4551 (6)	0.3542 (6)	0.3186 (5)	0.0414 (16)	
H12A	0.4176	0.3316	0.3838	0.050*	
H12B	0.5224	0.3181	0.3098	0.050*	
C4	0.2032 (6)	0.7055 (6)	−0.0929 (4)	0.0411 (16)	
H4A	0.1636	0.7514	−0.1346	0.049*	
H4B	0.1705	0.6199	−0.1189	0.049*	
C14	0.6484 (6)	0.8081 (6)	0.4222 (4)	0.0335 (14)	
H14A	0.7062	0.8568	0.4778	0.040*	
H14B	0.6522	0.7256	0.4213	0.040*	
C6	−0.0179 (5)	0.6034 (5)	0.2479 (5)	0.0353 (14)	
H6A	−0.0319	0.6093	0.1760	0.042*	
H6B	−0.1039	0.5624	0.2758	0.042*	
C3	0.3516 (6)	0.7598 (6)	−0.0988 (4)	0.0405 (16)	
H3A	0.3773	0.7455	−0.1660	0.049*	
H3B	0.3845	0.8486	−0.0832	0.049*	
C7	0.0525 (6)	0.6830 (6)	0.4735 (5)	0.0420 (16)	
H7A	0.0370	0.7080	0.5409	0.050*	
H7B	0.0083	0.5937	0.4643	0.050*	
C5	0.0676 (5)	0.5254 (5)	0.2648 (5)	0.0356 (14)	
H5A	0.0602	0.4979	0.3334	0.043*	
H5B	0.0367	0.4524	0.2192	0.043*	
O9	0.9349 (6)	0.8985 (7)	0.1774 (5)	0.093 (2)	
H9	0.8969	0.8687	0.1230	0.139*	
C15	0.6687 (6)	1.0703 (6)	0.3450 (5)	0.0432 (16)	
H15A	0.6339	1.0601	0.4120	0.052*	
H15B	0.7128	1.1584	0.3353	0.052*	
C11	0.3460 (7)	0.3024 (6)	0.2354 (5)	0.0480 (17)	
H11A	0.3850	0.3088	0.1701	0.058*	
H11B	0.2952	0.2155	0.2454	0.058*	
C17	1.0282 (9)	1.0283 (9)	0.1646 (9)	0.107 (4)	
H17A	0.9832	1.0850	0.1766	0.161*	
H17B	1.0587	1.0360	0.0972	0.161*	
H17C	1.1027	1.0476	0.2119	0.161*	

### Atomic displacement parameters (Å^2^)

**Table:**

	*U*^11^	*U*^22^	*U*^33^	*U*^12^	*U*^13^	*U*^23^
Ni1	0.0271 (4)	0.0258 (4)	0.0237 (4)	0.0097 (3)	−0.0053 (3)	0.0029 (3)
Ni2	0.0257 (4)	0.0264 (4)	0.0259 (4)	0.0104 (3)	−0.0030 (3)	0.0056 (3)
Ni3	0.0233 (4)	0.0267 (4)	0.0253 (4)	0.0095 (3)	−0.0015 (3)	0.0032 (3)
Ni4	0.0235 (4)	0.0268 (4)	0.0242 (4)	0.0079 (3)	−0.0019 (3)	0.0042 (3)
Cl4	0.0358 (8)	0.0429 (9)	0.0354 (9)	0.0092 (7)	0.0057 (6)	0.0124 (7)
Cl1	0.0437 (9)	0.0325 (8)	0.0408 (9)	0.0122 (7)	−0.0117 (7)	−0.0032 (6)
Cl3	0.0429 (9)	0.0321 (8)	0.0403 (9)	0.0155 (7)	0.0002 (7)	−0.0022 (6)
Cl2	0.0353 (8)	0.0430 (9)	0.0355 (9)	0.0148 (7)	0.0023 (6)	0.0154 (7)
O1	0.027 (2)	0.021 (2)	0.022 (2)	0.0098 (16)	−0.0031 (15)	0.0010 (15)
O3	0.0181 (18)	0.026 (2)	0.028 (2)	0.0089 (16)	−0.0026 (15)	0.0007 (15)
O7	0.0215 (19)	0.028 (2)	0.0189 (19)	0.0087 (16)	−0.0013 (14)	0.0032 (15)
O5	0.026 (2)	0.029 (2)	0.022 (2)	0.0116 (17)	−0.0022 (15)	0.0061 (15)
O4	0.035 (2)	0.041 (3)	0.037 (2)	0.015 (2)	0.0031 (18)	0.0088 (19)
O8	0.034 (2)	0.037 (2)	0.039 (2)	0.0086 (19)	−0.0036 (18)	−0.0017 (19)
O2	0.039 (2)	0.040 (3)	0.028 (2)	0.016 (2)	−0.0029 (17)	0.0030 (18)
N1	0.030 (3)	0.031 (3)	0.035 (3)	0.012 (2)	−0.006 (2)	0.007 (2)
N3	0.040 (3)	0.035 (3)	0.035 (3)	0.022 (2)	−0.008 (2)	0.001 (2)
C2	0.028 (3)	0.035 (3)	0.038 (4)	0.015 (3)	−0.005 (2)	0.008 (3)
C13	0.027 (3)	0.038 (3)	0.023 (3)	0.008 (3)	−0.001 (2)	0.005 (2)
O6	0.049 (3)	0.033 (2)	0.040 (3)	0.017 (2)	−0.009 (2)	−0.0011 (19)
C10	0.031 (3)	0.038 (4)	0.044 (4)	0.020 (3)	−0.006 (3)	0.001 (3)
C9	0.036 (3)	0.034 (3)	0.031 (3)	0.026 (3)	0.008 (2)	0.002 (2)
C1	0.034 (3)	0.027 (3)	0.027 (3)	0.016 (3)	−0.005 (2)	0.007 (2)
C16	0.035 (4)	0.042 (4)	0.048 (4)	0.002 (3)	−0.005 (3)	0.003 (3)
C8	0.029 (3)	0.044 (4)	0.043 (4)	0.019 (3)	0.010 (3)	0.005 (3)
N4	0.028 (3)	0.043 (3)	0.036 (3)	0.015 (2)	−0.001 (2)	0.004 (2)
N2	0.028 (3)	0.037 (3)	0.034 (3)	0.014 (2)	−0.001 (2)	0.010 (2)
C12	0.048 (4)	0.039 (4)	0.047 (4)	0.027 (3)	0.001 (3)	0.012 (3)
C4	0.053 (4)	0.046 (4)	0.027 (3)	0.022 (3)	−0.015 (3)	0.000 (3)
C14	0.035 (3)	0.043 (4)	0.025 (3)	0.018 (3)	−0.008 (2)	−0.003 (3)
C6	0.024 (3)	0.035 (4)	0.045 (4)	0.009 (3)	−0.006 (3)	0.004 (3)
C3	0.058 (4)	0.043 (4)	0.024 (3)	0.022 (3)	−0.001 (3)	0.007 (3)
C7	0.035 (4)	0.051 (4)	0.040 (4)	0.015 (3)	0.012 (3)	0.006 (3)
C5	0.025 (3)	0.030 (3)	0.044 (4)	0.001 (3)	−0.008 (3)	0.000 (3)
O9	0.086 (5)	0.126 (6)	0.090 (5)	0.066 (4)	−0.002 (4)	0.016 (4)
C15	0.047 (4)	0.034 (4)	0.039 (4)	0.005 (3)	−0.008 (3)	−0.006 (3)
C11	0.062 (5)	0.035 (4)	0.050 (4)	0.022 (3)	−0.005 (3)	0.001 (3)
C17	0.053 (6)	0.089 (8)	0.171 (12)	0.015 (5)	0.022 (6)	0.001 (7)

### Geometric parameters (Å, °)

**Table:**

Ni1—O1	2.050 (3)	O6—H6	0.8200
Ni1—O5	2.064 (4)	C10—C9	1.530 (8)
Ni1—N1	2.100 (4)	C10—H10A	0.9700
Ni1—O3	2.137 (4)	C10—H10B	0.9700
Ni1—O2	2.160 (4)	C9—H9A	0.9700
Ni1—Cl1	2.4081 (16)	C9—H9B	0.9700
Ni2—O5	2.044 (3)	C1—H1A	0.9700
Ni2—O3	2.052 (3)	C1—H1B	0.9700
Ni2—N3	2.112 (5)	C16—N4	1.489 (8)
Ni2—O7	2.117 (4)	C16—C15	1.511 (9)
Ni2—O6	2.168 (4)	C16—H16A	0.9700
Ni2—Cl2	2.4331 (16)	C16—H16B	0.9700
Ni3—O3	2.045 (4)	C8—N2	1.463 (7)
Ni3—O7	2.063 (3)	C8—C7	1.522 (8)
Ni3—O1	2.101 (3)	C8—H8A	0.9700
Ni3—N2	2.116 (4)	C8—H8B	0.9700
Ni3—O4	2.144 (4)	N4—C14	1.480 (7)
Ni3—Cl3	2.3888 (16)	N4—H4AA	0.9100
Ni4—O7	2.039 (3)	N2—C6	1.486 (7)
Ni4—O1	2.059 (3)	N2—H2AA	0.9100
Ni4—N4	2.123 (5)	C12—C11	1.529 (9)
Ni4—O5	2.133 (4)	C12—H12A	0.9700
Ni4—O8	2.145 (4)	C12—H12B	0.9700
Ni4—Cl4	2.3898 (16)	C4—C3	1.503 (9)
O1—C1	1.411 (6)	C4—H4A	0.9700
O3—C5	1.424 (6)	C4—H4B	0.9700
O7—C13	1.416 (6)	C14—H14A	0.9700
O5—C9	1.412 (6)	C14—H14B	0.9700
O4—C7	1.428 (7)	C6—C5	1.525 (8)
O4—H4	0.8200	C6—H6A	0.9700
O8—C15	1.438 (7)	C6—H6B	0.9700
O8—H8	0.8200	C3—H3A	0.9700
O2—C3	1.435 (7)	C3—H3B	0.9700
O2—H2	0.8200	C7—H7A	0.9700
N1—C4	1.485 (7)	C7—H7B	0.9700
N1—C2	1.471 (7)	C5—H5A	0.9700
N1—H1	0.9100	C5—H5B	0.9700
N3—C12	1.472 (7)	O9—C17	1.492 (10)
N3—C10	1.467 (7)	O9—H9	0.8200
N3—H3	0.9100	C15—H15A	0.9700
C2—C1	1.533 (7)	C15—H15B	0.9700
C2—H2A	0.9700	C11—H11A	0.9700
C2—H2B	0.9700	C11—H11B	0.9700
C13—C14	1.515 (7)	C17—H17A	0.9600
C13—H13A	0.9700	C17—H17B	0.9600
C13—H13B	0.9700	C17—H17C	0.9600
O6—C11	1.417 (7)		
			
O1—Ni1—O5	82.68 (14)	O7—C13—H13A	109.7
O1—Ni1—N1	83.49 (16)	C14—C13—H13A	109.7
O5—Ni1—N1	159.63 (17)	O7—C13—H13B	109.7
O1—Ni1—O3	78.70 (14)	C14—C13—H13B	109.7
O5—Ni1—O3	80.83 (13)	H13A—C13—H13B	108.2
N1—Ni1—O3	110.95 (16)	C11—O6—Ni2	113.3 (4)
O1—Ni1—O2	96.07 (14)	C11—O6—H6	109.5
O5—Ni1—O2	87.03 (14)	Ni2—O6—H6	66.2
N1—Ni1—O2	79.64 (16)	N3—C10—C9	112.0 (5)
O3—Ni1—O2	167.27 (14)	N3—C10—H10A	109.2
O1—Ni1—Cl1	170.51 (11)	C9—C10—H10A	109.2
O5—Ni1—Cl1	102.11 (11)	N3—C10—H10B	109.2
N1—Ni1—Cl1	93.83 (14)	C9—C10—H10B	109.2
O3—Ni1—Cl1	93.87 (10)	H10A—C10—H10B	107.9
O2—Ni1—Cl1	92.38 (11)	O5—C9—C10	109.6 (4)
O5—Ni2—O3	83.39 (14)	O5—C9—H9A	109.7
O5—Ni2—N3	83.37 (17)	C10—C9—H9A	109.7
O3—Ni2—N3	159.85 (17)	O5—C9—H9B	109.7
O5—Ni2—O7	79.11 (14)	C10—C9—H9B	109.7
O3—Ni2—O7	81.33 (13)	H9A—C9—H9B	108.2
N3—Ni2—O7	110.89 (16)	O1—C1—C2	108.8 (4)
O5—Ni2—O6	95.49 (15)	O1—C1—H1A	109.9
O3—Ni2—O6	86.76 (15)	C2—C1—H1A	109.9
N3—Ni2—O6	79.46 (17)	O1—C1—H1B	109.9
O7—Ni2—O6	167.40 (14)	C2—C1—H1B	109.9
O5—Ni2—Cl2	170.64 (11)	H1A—C1—H1B	108.3
O3—Ni2—Cl2	100.80 (11)	N4—C16—C15	111.5 (5)
N3—Ni2—Cl2	94.61 (14)	N4—C16—H16A	109.3
O7—Ni2—Cl2	93.16 (10)	C15—C16—H16A	109.3
O6—Ni2—Cl2	93.12 (12)	N4—C16—H16B	109.3
O3—Ni3—O7	82.80 (13)	C15—C16—H16B	109.3
O3—Ni3—O1	79.64 (14)	H16A—C16—H16B	108.0
O7—Ni3—O1	81.65 (13)	N2—C8—C7	110.4 (5)
O3—Ni3—N2	83.21 (16)	N2—C8—H8A	109.6
O7—Ni3—N2	158.91 (17)	C7—C8—H8A	109.6
O1—Ni3—N2	111.15 (16)	N2—C8—H8B	109.6
O3—Ni3—O4	97.16 (15)	C7—C8—H8B	109.6
O7—Ni3—O4	87.04 (14)	H8A—C8—H8B	108.1
O1—Ni3—O4	168.54 (14)	C16—N4—C14	115.2 (5)
N2—Ni3—O4	79.14 (16)	C16—N4—Ni4	107.8 (4)
O3—Ni3—Cl3	171.15 (11)	C14—N4—Ni4	107.2 (3)
O7—Ni3—Cl3	101.82 (11)	C16—N4—H4AA	108.8
O1—Ni3—Cl3	93.46 (10)	C14—N4—H4AA	108.8
N2—Ni3—Cl3	94.26 (14)	Ni4—N4—H4AA	108.8
O4—Ni3—Cl3	90.66 (12)	C8—N2—C6	115.5 (5)
O7—Ni4—O1	83.25 (14)	C8—N2—Ni3	107.0 (3)
O7—Ni4—N4	83.12 (16)	C6—N2—Ni3	107.9 (3)
O1—Ni4—N4	159.57 (17)	C8—N2—H2AA	108.7
O7—Ni4—O5	78.83 (14)	C6—N2—H2AA	108.7
O1—Ni4—O5	80.81 (13)	Ni3—N2—H2AA	108.7
N4—Ni4—O5	111.26 (17)	N3—C12—C11	110.6 (5)
O7—Ni4—O8	97.15 (15)	N3—C12—H12A	109.5
O1—Ni4—O8	87.13 (14)	C11—C12—H12A	109.5
N4—Ni4—O8	79.56 (17)	N3—C12—H12B	109.5
O5—Ni4—O8	167.63 (14)	C11—C12—H12B	109.5
O7—Ni4—Cl4	170.45 (11)	H12A—C12—H12B	108.1
O1—Ni4—Cl4	101.52 (11)	N1—C4—C3	111.3 (5)
N4—Ni4—Cl4	94.28 (14)	N1—C4—H4A	109.4
O5—Ni4—Cl4	93.67 (10)	C3—C4—H4A	109.4
O8—Ni4—Cl4	91.39 (12)	N1—C4—H4B	109.4
C1—O1—Ni4	124.9 (3)	C3—C4—H4B	109.4
C1—O1—Ni1	110.1 (3)	H4A—C4—H4B	108.0
Ni4—O1—Ni1	98.59 (14)	N4—C14—C13	111.1 (4)
C1—O1—Ni3	122.1 (3)	N4—C14—H14A	109.4
Ni4—O1—Ni3	95.45 (14)	C13—C14—H14A	109.4
Ni1—O1—Ni3	101.20 (15)	N4—C14—H14B	109.4
C5—O3—Ni3	109.6 (3)	C13—C14—H14B	109.4
C5—O3—Ni2	123.2 (3)	H14A—C14—H14B	108.0
Ni3—O3—Ni2	98.34 (14)	N2—C6—C5	112.1 (4)
C5—O3—Ni1	125.0 (3)	N2—C6—H6A	109.2
Ni3—O3—Ni1	100.17 (15)	C5—C6—H6A	109.2
Ni2—O3—Ni1	95.53 (14)	N2—C6—H6B	109.2
C13—O7—Ni4	109.8 (3)	C5—C6—H6B	109.2
C13—O7—Ni3	125.5 (3)	H6A—C6—H6B	107.9
Ni4—O7—Ni3	97.27 (14)	O2—C3—C4	106.3 (5)
C13—O7—Ni2	122.4 (3)	O2—C3—H3A	110.5
Ni4—O7—Ni2	101.27 (14)	C4—C3—H3A	110.5
Ni3—O7—Ni2	95.75 (14)	O2—C3—H3B	110.5
C9—O5—Ni2	109.5 (3)	C4—C3—H3B	110.5
C9—O5—Ni1	124.0 (3)	H3A—C3—H3B	108.7
Ni2—O5—Ni1	98.06 (15)	O4—C7—C8	107.8 (5)
C9—O5—Ni4	124.0 (3)	O4—C7—H7A	110.1
Ni2—O5—Ni4	100.56 (15)	C8—C7—H7A	110.1
Ni1—O5—Ni4	95.85 (14)	O4—C7—H7B	110.1
C7—O4—Ni3	114.0 (3)	C8—C7—H7B	110.1
C7—O4—H4	109.5	H7A—C7—H7B	108.5
Ni3—O4—H4	101.4	O3—C5—C6	109.7 (5)
C15—O8—Ni4	114.3 (4)	O3—C5—H5A	109.7
C15—O8—H8	109.5	C6—C5—H5A	109.7
Ni4—O8—H8	98.8	O3—C5—H5B	109.7
C3—O2—Ni1	113.2 (3)	C6—C5—H5B	109.7
C3—O2—H2	109.5	H5A—C5—H5B	108.2
Ni1—O2—H2	96.3	C17—O9—H9	109.5
C4—N1—C2	114.6 (5)	O8—C15—C16	107.7 (5)
C4—N1—Ni1	106.8 (3)	O8—C15—H15A	110.2
C2—N1—Ni1	107.4 (3)	C16—C15—H15A	110.2
C4—N1—H1	109.3	O8—C15—H15B	110.2
C2—N1—H1	109.3	C16—C15—H15B	110.2
Ni1—N1—H1	109.3	H15A—C15—H15B	108.5
C12—N3—C10	116.2 (5)	O6—C11—C12	108.3 (5)
C12—N3—Ni2	106.7 (4)	O6—C11—H11A	110.0
C10—N3—Ni2	107.6 (3)	C12—C11—H11A	110.0
C12—N3—H3	108.7	O6—C11—H11B	110.0
C10—N3—H3	108.7	C12—C11—H11B	110.0
Ni2—N3—H3	108.7	H11A—C11—H11B	108.4
N1—C2—C1	112.7 (4)	O9—C17—H17A	109.5
N1—C2—H2A	109.1	O9—C17—H17B	109.5
C1—C2—H2A	109.1	H17A—C17—H17B	109.5
N1—C2—H2B	109.1	O9—C17—H17C	109.5
C1—C2—H2B	109.1	H17A—C17—H17C	109.5
H2A—C2—H2B	107.8	H17B—C17—H17C	109.5
O7—C13—C14	109.9 (4)		
			
O7—Ni4—O1—C1	147.4 (4)	O3—Ni2—O5—Ni4	85.98 (15)
N4—Ni4—O1—C1	99.0 (6)	N3—Ni2—O5—Ni4	−109.24 (18)
O5—Ni4—O1—C1	−132.9 (4)	O7—Ni2—O5—Ni4	3.58 (13)
O8—Ni4—O1—C1	49.9 (4)	O6—Ni2—O5—Ni4	172.07 (15)
Cl4—Ni4—O1—C1	−41.0 (4)	O1—Ni1—O5—C9	−149.1 (4)
O7—Ni4—O1—Ni1	−90.62 (15)	N1—Ni1—O5—C9	−101.5 (6)
N4—Ni4—O1—Ni1	−139.1 (4)	O3—Ni1—O5—C9	131.2 (4)
O5—Ni4—O1—Ni1	−10.90 (14)	O2—Ni1—O5—C9	−52.6 (4)
O8—Ni4—O1—Ni1	171.83 (16)	Cl1—Ni1—O5—C9	39.2 (4)
Cl4—Ni4—O1—Ni1	81.00 (13)	O1—Ni1—O5—Ni2	90.81 (15)
O7—Ni4—O1—Ni3	11.61 (14)	N1—Ni1—O5—Ni2	138.4 (4)
N4—Ni4—O1—Ni3	−36.8 (5)	O3—Ni1—O5—Ni2	11.15 (14)
O5—Ni4—O1—Ni3	91.33 (14)	O2—Ni1—O5—Ni2	−172.67 (16)
O8—Ni4—O1—Ni3	−85.94 (15)	Cl1—Ni1—O5—Ni2	−80.87 (13)
Cl4—Ni4—O1—Ni3	−176.77 (9)	O1—Ni1—O5—Ni4	−10.76 (14)
O5—Ni1—O1—C1	143.4 (3)	N1—Ni1—O5—Ni4	36.9 (5)
N1—Ni1—O1—C1	−21.6 (3)	O3—Ni1—O5—Ni4	−90.42 (14)
O3—Ni1—O1—C1	−134.6 (3)	O2—Ni1—O5—Ni4	85.76 (15)
O2—Ni1—O1—C1	57.2 (3)	Cl1—Ni1—O5—Ni4	177.56 (9)
O5—Ni1—O1—Ni4	11.22 (14)	O7—Ni4—O5—C9	−126.0 (4)
N1—Ni1—O1—Ni4	−153.78 (18)	O1—Ni4—O5—C9	149.1 (4)
O3—Ni1—O1—Ni4	93.28 (15)	N4—Ni4—O5—C9	−48.0 (4)
O2—Ni1—O1—Ni4	−74.98 (16)	O8—Ni4—O5—C9	162.0 (6)
O5—Ni1—O1—Ni3	−86.13 (15)	Cl4—Ni4—O5—C9	48.0 (4)
N1—Ni1—O1—Ni3	108.87 (18)	O7—Ni4—O5—Ni2	−3.72 (14)
O3—Ni1—O1—Ni3	−4.07 (13)	O1—Ni4—O5—Ni2	−88.59 (15)
O2—Ni1—O1—Ni3	−172.33 (15)	N4—Ni4—O5—Ni2	74.29 (19)
O3—Ni3—O1—C1	126.8 (4)	O8—Ni4—O5—Ni2	−75.7 (7)
O7—Ni3—O1—C1	−149.0 (4)	Cl4—Ni4—O5—Ni2	170.32 (12)
N2—Ni3—O1—C1	48.3 (4)	O7—Ni4—O5—Ni1	95.63 (15)
O4—Ni3—O1—C1	−158.5 (7)	O1—Ni4—O5—Ni1	10.76 (14)
Cl3—Ni3—O1—C1	−47.6 (4)	N4—Ni4—O5—Ni1	173.64 (16)
O3—Ni3—O1—Ni4	−95.66 (14)	O8—Ni4—O5—Ni1	23.6 (7)
O7—Ni3—O1—Ni4	−11.51 (14)	Cl4—Ni4—O5—Ni1	−90.33 (11)
N2—Ni3—O1—Ni4	−174.14 (15)	O3—Ni3—O4—C7	−81.7 (4)
O4—Ni3—O1—Ni4	−21.0 (8)	O7—Ni3—O4—C7	−164.1 (4)
Cl3—Ni3—O1—Ni4	89.94 (11)	O1—Ni3—O4—C7	−154.7 (6)
O3—Ni3—O1—Ni1	4.24 (14)	N2—Ni3—O4—C7	−0.1 (4)
O7—Ni3—O1—Ni1	88.39 (15)	Cl3—Ni3—O4—C7	94.1 (4)
N2—Ni3—O1—Ni1	−74.24 (19)	O7—Ni4—O8—C15	77.5 (4)
O4—Ni3—O1—Ni1	78.9 (7)	O1—Ni4—O8—C15	160.3 (4)
Cl3—Ni3—O1—Ni1	−170.16 (12)	N4—Ni4—O8—C15	−4.1 (4)
O7—Ni3—O3—C5	140.1 (3)	O5—Ni4—O8—C15	147.6 (6)
O1—Ni3—O3—C5	−137.1 (3)	Cl4—Ni4—O8—C15	−98.2 (4)
N2—Ni3—O3—C5	−24.1 (3)	O1—Ni1—O2—C3	−77.4 (4)
O4—Ni3—O3—C5	54.0 (3)	O5—Ni1—O2—C3	−159.7 (4)
O7—Ni3—O3—Ni2	10.35 (14)	N1—Ni1—O2—C3	4.8 (4)
O1—Ni3—O3—Ni2	93.13 (15)	O3—Ni1—O2—C3	−142.3 (6)
N2—Ni3—O3—Ni2	−153.84 (18)	Cl1—Ni1—O2—C3	98.3 (4)
O4—Ni3—O3—Ni2	−75.73 (16)	O1—Ni1—N1—C4	119.8 (4)
O7—Ni3—O3—Ni1	−86.84 (14)	O5—Ni1—N1—C4	72.3 (6)
O1—Ni3—O3—Ni1	−4.06 (13)	O3—Ni1—N1—C4	−165.0 (3)
N2—Ni3—O3—Ni1	108.97 (17)	O2—Ni1—N1—C4	22.4 (4)
O4—Ni3—O3—Ni1	−172.92 (14)	Cl1—Ni1—N1—C4	−69.3 (4)
O5—Ni2—O3—C5	149.8 (4)	O1—Ni1—N1—C2	−3.6 (3)
N3—Ni2—O3—C5	100.6 (6)	O5—Ni1—N1—C2	−51.1 (7)
O7—Ni2—O3—C5	−130.2 (4)	O3—Ni1—N1—C2	71.6 (4)
O6—Ni2—O3—C5	53.9 (4)	O2—Ni1—N1—C2	−101.0 (4)
Cl2—Ni2—O3—C5	−38.6 (4)	Cl1—Ni1—N1—C2	167.3 (3)
O5—Ni2—O3—Ni3	−90.06 (15)	O5—Ni2—N3—C12	−123.0 (4)
N3—Ni2—O3—Ni3	−139.3 (4)	O3—Ni2—N3—C12	−73.8 (6)
O7—Ni2—O3—Ni3	−10.12 (14)	O7—Ni2—N3—C12	161.4 (3)
O6—Ni2—O3—Ni3	174.03 (16)	O6—Ni2—N3—C12	−26.1 (4)
Cl2—Ni2—O3—Ni3	81.47 (13)	Cl2—Ni2—N3—C12	66.2 (4)
O5—Ni2—O3—Ni1	11.09 (14)	O5—Ni2—N3—C10	2.4 (3)
N3—Ni2—O3—Ni1	−38.1 (5)	O3—Ni2—N3—C10	51.7 (7)
O7—Ni2—O3—Ni1	91.03 (14)	O7—Ni2—N3—C10	−73.2 (4)
O6—Ni2—O3—Ni1	−84.82 (15)	O6—Ni2—N3—C10	99.3 (4)
Cl2—Ni2—O3—Ni1	−177.38 (9)	Cl2—Ni2—N3—C10	−168.4 (3)
O1—Ni1—O3—C5	127.0 (4)	C4—N1—C2—C1	−91.7 (6)
O5—Ni1—O3—C5	−148.7 (4)	Ni1—N1—C2—C1	26.7 (5)
N1—Ni1—O3—C5	48.5 (4)	Ni4—O7—C13—C14	−42.6 (5)
O2—Ni1—O3—C5	−166.3 (6)	Ni3—O7—C13—C14	−157.5 (3)
Cl1—Ni1—O3—C5	−47.1 (4)	Ni2—O7—C13—C14	75.8 (5)
O1—Ni1—O3—Ni3	4.17 (13)	O5—Ni2—O6—C11	82.6 (4)
O5—Ni1—O3—Ni3	88.47 (15)	O3—Ni2—O6—C11	165.6 (4)
N1—Ni1—O3—Ni3	−74.28 (19)	N3—Ni2—O6—C11	0.4 (4)
O2—Ni1—O3—Ni3	70.9 (7)	O7—Ni2—O6—C11	146.5 (6)
Cl1—Ni1—O3—Ni3	−169.87 (11)	Cl2—Ni2—O6—C11	−93.8 (4)
O1—Ni1—O3—Ni2	−95.35 (15)	C12—N3—C10—C9	93.9 (6)
O5—Ni1—O3—Ni2	−11.05 (14)	Ni2—N3—C10—C9	−25.6 (5)
N1—Ni1—O3—Ni2	−173.80 (16)	Ni2—O5—C9—C10	−42.5 (5)
O2—Ni1—O3—Ni2	−28.6 (7)	Ni1—O5—C9—C10	−157.2 (3)
Cl1—Ni1—O3—Ni2	90.60 (11)	Ni4—O5—C9—C10	75.7 (5)
O1—Ni4—O7—C13	−143.7 (3)	N3—C10—C9—O5	46.3 (6)
N4—Ni4—O7—C13	21.0 (3)	Ni4—O1—C1—C2	157.8 (3)
O5—Ni4—O7—C13	134.4 (3)	Ni1—O1—C1—C2	41.1 (5)
O8—Ni4—O7—C13	−57.5 (3)	Ni3—O1—C1—C2	−77.2 (5)
O1—Ni4—O7—Ni3	−11.87 (14)	N1—C2—C1—O1	−46.1 (6)
N4—Ni4—O7—Ni3	152.88 (18)	C15—C16—N4—C14	−75.9 (6)
O5—Ni4—O7—Ni3	−93.78 (15)	C15—C16—N4—Ni4	43.7 (6)
O8—Ni4—O7—Ni3	74.37 (16)	O7—Ni4—N4—C16	−119.7 (4)
O1—Ni4—O7—Ni2	85.51 (15)	O1—Ni4—N4—C16	−71.3 (6)
N4—Ni4—O7—Ni2	−109.74 (18)	O5—Ni4—N4—C16	165.1 (3)
O5—Ni4—O7—Ni2	3.60 (13)	O8—Ni4—N4—C16	−21.1 (4)
O8—Ni4—O7—Ni2	171.75 (15)	Cl4—Ni4—N4—C16	69.5 (4)
O3—Ni3—O7—C13	−147.1 (4)	O7—Ni4—N4—C14	4.9 (3)
O1—Ni3—O7—C13	132.3 (4)	O1—Ni4—N4—C14	53.3 (7)
N2—Ni3—O7—C13	−98.4 (6)	O5—Ni4—N4—C14	−70.3 (4)
O4—Ni3—O7—C13	−49.5 (4)	O8—Ni4—N4—C14	103.5 (4)
Cl3—Ni3—O7—C13	40.5 (4)	Cl4—Ni4—N4—C14	−165.9 (3)
O3—Ni3—O7—Ni4	92.20 (15)	C7—C8—N2—C6	71.9 (6)
O1—Ni3—O7—Ni4	11.67 (14)	C7—C8—N2—Ni3	−48.3 (5)
N2—Ni3—O7—Ni4	141.0 (4)	O3—Ni3—N2—C8	124.7 (4)
O4—Ni3—O7—Ni4	−170.20 (15)	O7—Ni3—N2—C8	76.0 (6)
Cl3—Ni3—O7—Ni4	−80.14 (12)	O1—Ni3—N2—C8	−159.2 (3)
O3—Ni3—O7—Ni2	−9.97 (13)	O4—Ni3—N2—C8	26.0 (4)
O1—Ni3—O7—Ni2	−90.50 (14)	Cl3—Ni3—N2—C8	−63.8 (4)
N2—Ni3—O7—Ni2	38.8 (5)	O3—Ni3—N2—C6	−0.2 (4)
O4—Ni3—O7—Ni2	87.63 (15)	O7—Ni3—N2—C6	−48.9 (6)
Cl3—Ni3—O7—Ni2	177.69 (9)	O1—Ni3—N2—C6	75.9 (4)
O5—Ni2—O7—C13	−126.1 (4)	O4—Ni3—N2—C6	−98.9 (4)
O3—Ni2—O7—C13	149.0 (4)	Cl3—Ni3—N2—C6	171.2 (3)
N3—Ni2—O7—C13	−47.6 (4)	C10—N3—C12—C11	−71.7 (6)
O6—Ni2—O7—C13	168.3 (6)	Ni2—N3—C12—C11	48.3 (6)
Cl2—Ni2—O7—C13	48.5 (3)	C2—N1—C4—C3	71.2 (6)
O5—Ni2—O7—Ni4	−3.75 (14)	Ni1—N1—C4—C3	−47.6 (6)
O3—Ni2—O7—Ni4	−88.63 (15)	C16—N4—C14—C13	91.4 (6)
N3—Ni2—O7—Ni4	74.76 (19)	Ni4—N4—C14—C13	−28.6 (5)
O6—Ni2—O7—Ni4	−69.3 (7)	O7—C13—C14—N4	48.2 (6)
Cl2—Ni2—O7—Ni4	170.92 (12)	C8—N2—C6—C5	−96.5 (6)
O5—Ni2—O7—Ni3	94.86 (15)	Ni3—N2—C6—C5	23.2 (6)
O3—Ni2—O7—Ni3	9.98 (13)	Ni1—O2—C3—C4	−30.4 (5)
N3—Ni2—O7—Ni3	173.37 (16)	N1—C4—C3—O2	51.8 (6)
O6—Ni2—O7—Ni3	29.3 (7)	Ni3—O4—C7—C8	−24.9 (6)
Cl2—Ni2—O7—Ni3	−90.47 (11)	N2—C8—C7—O4	48.8 (7)
O3—Ni2—O5—C9	−142.0 (3)	Ni3—O3—C5—C6	43.0 (5)
N3—Ni2—O5—C9	22.8 (3)	Ni2—O3—C5—C6	157.7 (4)
O7—Ni2—O5—C9	135.6 (3)	Ni1—O3—C5—C6	−75.5 (5)
O6—Ni2—O5—C9	−55.9 (3)	N2—C6—C5—O3	−44.6 (7)
O3—Ni2—O5—Ni1	−11.55 (14)	Ni4—O8—C15—C16	27.9 (6)
N3—Ni2—O5—Ni1	153.23 (19)	N4—C16—C15—O8	−47.4 (7)
O7—Ni2—O5—Ni1	−93.95 (15)	Ni2—O6—C11—C12	24.7 (6)
O6—Ni2—O5—Ni1	74.54 (16)	N3—C12—C11—O6	−48.9 (7)

### Hydrogen-bond geometry (Å, °)

**Table:**

*D*—H···*A*	*D*—H	H···*A*	*D*···*A*	*D*—H···*A*
O4—H4···Cl2	0.82	2.27	3.087 (4)	176
O8—H8···Cl3	0.82	2.19	3.013 (4)	179
O2—H2···Cl4	0.82	2.24	3.058 (4)	175
O6—H6···Cl2	0.82	2.60	3.346 (4)	153
N1—H1···Cl1^i^	0.91	2.62	3.476 (5)	157
N3—H3···Cl2^ii^	0.91	2.62	3.448 (5)	152
N4—H4AA···O9	0.91	2.52	3.228 (9)	135
N2—H2AA···O9^iii^	0.91	2.18	3.060 (10)	162
O9—H9···Cl4	0.82	2.76	3.224 (7)	118

Symmetry codes: (i) −*x*, −*y*+1, −*z*; (ii) −*x*+1, −*y*+1, −*z*+1; (iii) *x*−1, *y*, *z*.
